# Risk profiles of prostate cancers identified from UK primary care using national referral guidelines

**DOI:** 10.1038/bjc.2011.596

**Published:** 2012-01-12

**Authors:** H Serag, S Banerjee, K Saeb-Parsy, S Irving, K Wright, S Stearn, A Doble, V J Gnanapragasam

**Affiliations:** 1 Department of Urology, Addenbrookes Hospital, Hills Road, Cambridge CB2 0XZ, UK; 2 Department of Urology, Norfolk and Norwich Hospital, Colney Lane, Norwich NR4 7UY, UK; 3 Eastern Cancer Registry and Information Centre, Cambridge CB22 3AD, UK; 4 Translational Prostate Cancer Group, Hutchison/MRC Research Centre, University of Cambridge, Hills Road, Cambridge CB1 0XZ, UK

**Keywords:** prostate cancer, risk profiles, Cancer Referral Guidelines, detection

## Abstract

**Objective:**

Prostate cancer in the United Kingdom is mainly diagnosed from primary care referrals based on national guidelines published by the Department of Health. Here we investigated the characteristics of cancers detected through the use of these guidelines.

**Methods:**

A prospective two-centre study was established to assess men referred from the primary care based on the UK national guidelines.

**Results:**

The overall cancer detection rate was 43% (169 out of 397) with 15% (26 out of 169) of all cancers metastatic at presentation. Amongst 50–69-year-old men these rates were 34% (68 out of 200) and 15% (10 out of 68). Only 21% (25 out of 123) of men with local cancers had low-risk disease. In comparison to a historical cohort from 2001 (*n*=137) we found no overall differences in rates of metastatic disease, locally advanced tumours, or risk categories. Amongst 50–69-year-old men with local disease, however, we observed an increase in detection of low-risk cancers in a contemporary cohort (*P*=0.04). This was primarily because of the increased detection of low-stage organ-confined tumours in this group (*P*=0.02).

**Conclusion:**

Use of the UK prostate cancer guidelines detects a high proportion of clinically significant cancers. Use of the guidelines does not seem to have led to an overall change in the clinical characteristics of presenting cancers. There may, however, be a specific benefit in detecting more low-risk disease in younger men.

Prostate cancer is the most common malignancy of men in the United Kingdom and a leading cause of cancer related death. Screening for prostate cancer using the PSA test has been proposed as a means of increasing early detection and reducing mortality. This, however, comes at the cost of significant over-detection with many men diagnosed with low-risk clinically insignificant disease (Schröder *et al*, 2009; Sandblom *et al*, 2011). At the present time there is no screening programme in the United Kingdom for prostate cancer and this is unlikely to be introduced with the currently available PSA test as a tool (Neal *et al*, 2009).

In the United Kingdom, general practitioners (GP) are the first physicians to see patients with suspected prostate cancer. Ten years ago, the Department of Health (DOH) implemented the National Health Service (NHS) cancer plan, which led to introduction of guidelines to aid GP referrals to tertiary centres for suspected cancers (Department of Health, The NHS cancer plan, 2000). These guidelines were further updated in 2005 following reviews and audits (National Institute for Health and Clinical Excellence, 2005). In response to these guidelines many hospitals established rapid access and one stop prostate diagnostic clinics to see these patients. This mechanism is currently the main pathway for testing and detection of the prostate cancer in the United Kingdom. In this study, our aim was to investigate the presenting characteristics and risk categorisation of prostate cancers diagnosed using the current version (2005) of the UK national guidelines.

## Patients and methods

A prospective study was set up in two tertiary centres both of which undertake prostate cancer diagnostic clinics to see patients referred under the DOH urgent cancer referral guidelines. The study was performed over two separate time periods and all GP referrals to these clinics in the time frame were included. Data collection was prospectively undertaken in Hospital A (*n*=272, Addenbrookes Hospital, Cambridge; June–December, 2009) and Hospital B (*n*=125, Norfolk and Norwich NHS trust; May—August, 2010). As this study focused on referrals through the guidelines, diagnosis through other pathways were excluded. A prostate cancer diagnosis was made based on the biopsy-proven tumour or a high index of clinical suspicion warranting initiation of androgen deprivation therapy. Non-cancer patients were followed for 12 months after the first clinic appointment to ensure that no subsequent prostate cancer diagnosis was made within 1 year. Disease parameters were determined for the group as a whole and then also defined *a priori* separately for the 50–69-year group separately. This was done to specifically assess the impact of these guidelines on cancer-risk groups in the potential screening age population as defined by recently published large screening trials (Schröder *et al*, 2009; Sandblom *et al*, 2011). All data were unified in a single database and centrally analysed by an independent assessor (VG). Risk categories were defined by the NICE published guidelines for localised prostate cancer. Comparison of tumour characteristics and risk categorisation in Hospital A (*n*=103) was then made with a historical cohort of patients seen in the prostate diagnostic clinics from the same hospital but before introduction of the 2005 version of the guidelines. To achieve this, cancer registry data from the Eastern Cancer Registry and Information Centre was reviewed for all prostate cancers diagnosed in 2001 in Hospital A and seen only in the prostate diagnostic clinics (*n*=137). Men diagnosed through any other routes were again excluded from this historical analysis cohort. To facilitate comparison between the groups, data on disease parameters were standardised for the fifth edition of the TNM criteria (AJCC comparison guide, fifth *vs* sixth edition, AJCC, http://www.cancerstaging.org/products/ajccguide.pdf). Statistical analysis was performed with Stats Direct software (Cheshire, UK) using logistic regression, chi-square, and the Mann–Whitney test.

## Results

### Tumour characteristics and risk stratification of detected cancers

In all, 169 out of 397 (43%) of the referred men were diagnosed with prostate cancer from the initial primary care referral (137 by biopsy and 32 by clinical diagnosis). Out of the 169 patients with cancer, 26 (15%) had evidence of bone metastasis at the time of diagnosis (Table 1). A total of 96 out of 169 (55%) had clinical stage T1–T2 (localised disease) whereas 61 out of 169 (38%) had T3–T4 (locally advanced disease). Data on stage were not available in a remaining 12 men. Gleason sum score was assessable in the 137 men who had a diagnostic biopsy (Table 1). There was an even spread of Gleason grades with 30% of men with Gleason sum 6 and Gleason 8–10 disease. The remaining men had Gleason 7 cancers. A similar analysis was next done focusing on the 50–69-year-old age group. Here, 68 out of 200 (34%) of the men were diagnosed with prostate cancer with the majority 61 out of 68 (91%) diagnosed from prostate biopsies. In this group a similar proportion of men had metastasis at diagnosis (10 out of 68, 15%). The majority had clinically localised disease 49 out of 68 (72%) with 16 out of 68 (28%) with locally advanced disease. When stratified by grade (*n*=62), 23 out of 62 (37%) had Gleason sum 6 disease, 26 out of 62 (42%) had Gleason 7 disease, and a minority, 13 out of 62 (21%), had Gleason sum 8–10 disease. We next assessed the risk categories of men presenting with local disease by stratifying men according to NICE-defined risk groups. Men who were clinically diagnosed or had metastasis were necessarily excluded from this analysis (Table 1). In the evaluated whole cohort (*n*=123) the majority of men were categorised as either intermediate or high risk, 50 out of 123 (41%) and 48 out of 123 (39%), respectively. Only 25 out of 123 (21%) of patients were low risk. There was no significant difference in the proportions of men in each risk group when comparison was made between the two centres (*P*=0.47, data not shown). In the 50–69-year cohort (*n*=58) there were, however, comparatively higher numbers of men with low-risk disease, 21 out of 58 (36%). A majority of men, however, still had intermediate-risk disease, 24 out of 58 (41%) and the smallest group was those with high-risk disease, 13 out of 58 (22%). There was again no difference in risk group distribution in comparison of the two centres (*P*=0.73).

### Comparison of tumour characteristics with a historical cohort

We next tested if the guidelines had altered the characteristics of referred tumours over a period of establishment and familiarity, and following the revision in 2005 (National Institute for Health and Clinical Excellence, 2005). These updated guidelines provided more detailed recommendations compared with the original guidelines, which only recommended referral for an elevated age-specific PSA or a PSA >20 in the presence of a malignant feeling gland or bone pain. The contemporary cohort of men diagnosed in hospital A were included in this analysis (*n*=103) and contrasted with a historical cohort from the same hospital. In this comparison (Table 2), we found no differences in the rates of metastasis (18% *vs* 23%) or clinically advanced disease at presentation (21% *vs* 15%) (Table 2). We also found no differences in the risk categories, Gleason sum scores, clinical stage, or median presenting PSA between the two cohorts when biopsy diagnosed men were compared. We then separately considered cancers detected in the 50–69-year age group in Hospital A between the contemporary and historical cohorts (Table 3). There was again no significant difference in metastasis rates or clinically advanced disease at presentation. Biopsy diagnosed men were next assessed for the risk type and tumour characteristics. Assessed by risk categories 23 out of 44 (52%) of men in the 2001 cohort had high-risk disease whereas only 10 out of 44 (23%) had low-risk disease. In the 2009 cohort, however, this trend was reversed with 15 out of 35 (43%) of men with low-risk disease and 9 out of 35 (26%) with high-risk disease (*P*=0.04) (Table 3). The percentage of men with intermediate-risk cancers was similar between the two cohorts. Analysis by Gleason sum score did not identify any significant change in the distribution amongst different cohorts (*P*=0.9). Similarly, there was no difference seen in the median PSA at diagnosis between cohorts (*P*=0.1). The most significant difference, however, was an apparent stage migration in the contemporary study cohort. Here, 13 out of 44 (30%) of tumours in the historical cohort were classed as locally advanced whereas only 4 out of 35 (11%) of the contemporary cohort were locally advanced at diagnosis (*P*=0.02) (Table 3).

## Interpretation

The intention of this study was to profile the nature of cancers detected through the national referral guidelines for GP's published by the UK DOH. In particular the incidence of metastasis, clinically advanced disease and in localised disease the risk categories of cancers. These factors are key determinants of the treatment needs of the patients. Here we found a high overall incidence of cancer detection of 43%. These rates, however, are very similar to data from other centres. Most studies have shown that while detection of all urological cancers is very variable, there is a consistently high rate of prostate cancers diagnosed from GP referrals. Hawary *et al* (2008) reviewed 170 patients referred for suspected urological cancer. In this series, 28% of patients were found to have cancer including 44% of men referred specifically for suspected prostate cancer. Mathew and Desai (2009) similarly reported on 400 suspected urological cancer referrals. The authors reported an overall cancer detection and prostate cancer-specific detection rate of 23% and 43%, respectively. Allgar *et al* (2006) found a 50% detection rate for all urgent referrals with suspected prostate cancer in one large district hospital trust over a 2-year period. These data suggest that use of the current guidelines results in a high rate of prostate cancer detection.

In this study 15% of diagnosed cancers had evidence of metastasis at presentation. In men with localised disease only a minority (20%) had low-risk prostate cancer whereas the majority had intermediate- or high-risk disease that warranted treatment. A key group for cancer detection is the 50–69-year-old demographic the so called ‘screening age group’ (Andriole *et al*, 2009; Schröder *et al*, 2009). Here, amongst men with localised disease the majority still had intermediate- or high-risk disease though a proportionally higher number (one in three) had low-risk disease. These results suggest that under the current guidelines the majority of detected cancers are clinically significant and warrant active treatment. Low risk and indolent is under-represented regardless of the age group of the patients.

Important reasons for introduction of the guidelines under the NHS cancer plan were to reduce the times from cancer diagnosis to treatment and to improve early detection rates. This study could not comment on any change in times to the prostate cancer treatment but did ask if more early cancers were being diagnosed. In comparison with a historical cohort studied in one centre, we did not observe any appreciable overall difference in the presenting tumours in terms of the numbers of men with metastasis or clinically diagnosed cancers. There was also no difference in tumour histopathological characteristics or overall risk categories. We acknowledge the weakness that the historical data were collected retrospectively whereas the contemporary data were prospective. In this context there could be several sources of bias, including a greater awareness of the prostate cancer, increased serendipitous PSA testing, or earlier presentation of men with lower urinary tract symptoms. Within the limitations of these possible biases, our findings, however, are similar to published reports in other tumours, which have also failed to show improvements in early detection through nationally implemented cancer guidelines (Debnath *et al*, 2002; Neal *et al*, 2007; McKie *et al*, 2008; Pencavel *et al*, 2010). In the present study, however, we did find that in the 50–69-year age group there was a significant trend towards detection of more organ confined tumours and as a consequence an apparent increase in the proportion of low-risk cancers.

To our knowledge this is the first prospective study to assess the tumour characteristics of prostate cancers detected through the UK national cancer referral guidelines. Our results suggest that the use of the guidelines have not led to any overall improvement in early detection of prostate cancer and most men will present with advanced disease or disease that warrants active treatment. Within the limits of our small cohort size there is preliminary evidence, however, that younger men may benefit most from these guidelines with increased proportions of men with low-risk disease because of a stage migration effect.

As the introduction of a screening programme in the United Kingdom is not imminent, GP referrals based on these guidelines will continue to be the primary route for men to be identified for investigation of suspected prostate cancer. Based on these preliminary results we do not currently advocate any change in this practise. Indeed the current guidelines do lead to a high rate of cancer detection as shown in this and other publications. These findings, however, do support the notion of further exploration of the risk profiles of presenting cancers by undertaking multi-centre prospective studies evaluating the cancer characteristics detected through urgent cancer referrals and stratified by age. This could then lead to an informed discussion of how the guidelines can be improved or perhaps focused on particular patient age groups.

## Figures and Tables

**Table 1:** Characteristics of cancers and risk categories detected in the whole cohort and in the 50–69-year age group (potential screening age)

	**Whole cohort (%)**	**50–69 year group (%)**
Total cancers detected	169	68
Clinical diagnosis	32 (19)	6 (9)
Biopsy diagnosis	137 (81)	62 (91)
Bone metastasis	26 (15)	10 (15)
		
*Gleason sum score*	(*n*=137)	(*n*=62)
⩽6	41 (30)	23 (37)
7	56 (40)	26 (42)
8–10	40 (30)	13 (21)
		
*Clinical stage*		
T1–T2	96 (55)	49 (72)
T3–T4	61 (38)	16 (23)
NA	12 (7)	3 (5)
		
*Risk group (localised cancers)*	(*n*=123)	(*n*=58)
Low	25 (20)	21 (36)
Intermediate	50 (41)	24 (41)
High	48 (39)	13 (22)

Clinical stage-TNM criteria.

**Table 2:** Comparison of characteristics of detected cancers in the prostate diagnostic clinics between the current cohort and a historical cohort from 2001 in Hospital A for men of all ages

	**2009 cohort (%) (*n*=103)**	**2001 cohort (%) (*n*=137)**	***P*-value**
Metastasis	19 (18)	31 (23)	0.4
Clinical diagnosis	22 (21)	21 (15)	0.2
			
*Risk group (localised cancers)*	(*n*=74)	(*n*=100)	
Low	21 (28)	22 (22)	
Intermediate	18 (24)	25 (25)	
High	35 (48)	53 (53)	0.6
			
*Gleason sum*			
⩽6	33 (45)	48 (48)	
7	21 (28)	29 (29)	
8–10	20 (27)	23 (23)	0.1
			
*Clinical stage*			
T1–T2	58 (78)	74 (74)	
T3–T4	16 (22)	26 (26)	0.5
			
*PSA μg l* ^*−1*^			
Median (range)	10.6 (2.8–82)	11.4 (3.9–424)	0.1

*P*<0.05 taken as statistically significant.

**Table 3:** Comparison of characteristics of detected cancers in the prostate diagnostic clinics between the current cohort and a historical cohort from 2001 in Hospital A for men aged 50–69 years

	**2009 cohort (%) (*n*=43)**	**2001 cohort (%) (*n*=52)**	***P*-value**
Metastasis	8 (18)	7 (13)	0.7
Clinical diagnosis	4 (9)	1 (2)	0.1
			
*Risk group (localised cancers)*	(*n*=35)	(*n*=44)	
Low	15 (43)	10 (23)	
Intermediate	11 (31)	11 (25)	
High	9 (26)	23 (52)	0.04
			
*Gleason sum*			
⩽6	18 (51)	23 (52)	
7	10 (29)	13 (30)	
8–10	7 (20)	8 (18)	0.9
			
*Clinical stage*			
T1–T2	31 (89)	31 (70)	
T3–T4	4 (11)	13 (30)	0.02
			
*PSA μg l* ^*−1*^			
Median (range)	7.7 (2.8–45.8)	10.5 (3.9–201)	0.1

*P*<0.05 taken as statistically significant.

## References

[bib1] Allgar VL, Neal RD, Ali N, Leese B, Heywood P, Proctor G, Evans J (2006) Urgent GP referrals for suspected lung, colorectal, prostate and ovarian cancer. Br J Gen Pract 56: 355–362 16638251 PMC1837844

[bib2] Andriole GL, Crawford ED, Grubb III RL, Buys SS, Chia D, Church TR, Fouad MN, Gelmann EP, Kvale PA, Reding DJ, Weissfeld JL, Yokochi LA, O’Brien B, Clapp JD, Rathmell JM, Riley TL, Hayes RB, Kramer BS, Izmirlian G, Miller AB, Pinsky PF, Prorok PC, Gohagan JK, Berg CD (2009) Mortality results from a randomized prostate-cancer screening trial. N Engl J Med 360: 1310–1319 19297565 10.1056/NEJMoa0810696 PMC2944770

[bib3] Debnath D, Dielehner N, Gunning KA (2002) Guidelines, compliance, and effectiveness: a 12 months’ audit in an acute district general healthcare trust on the two week rule for suspected colorectal cancer. Postgrad Med J 78: 748–751 12509694 10.1136/pmj.78.926.748 PMC1757929

[bib4] Department of Health. The NHS Cancer Plan. Department of Health: London, 2000

[bib5] Hawary AM, Warburton HE, Brough RJ, Collins GN, Brown SC, O’Reilly PH, Adeyoju AA (2008) The ‘2-week wait’ rule for referrals for suspected urological cancers--urgent need for refinement of criteria. Ann R Coll Surg Engl 90: 517–522 18765032 10.1308/003588408X301082 PMC2647249

[bib6] Mathew A, Desai KM (2009) An audit of urology two-week wait referrals in a large teaching hospital in England. Ann R Coll Surg Engl 91: 310–312 19344552 10.1308/003588409X391767 PMC2749401

[bib7] McKie C, Ahmad UA, Fellows S, Meikle D, Stafford FW, Thomson PJ, Welch AR, Paleri V (2008) The 2-week rule for suspected head and neck cancer in the United Kingdom: referral patterns, diagnostic efficacy of the guidelines and compliance. Oral Oncol 44: 851–856 18234546 10.1016/j.oraloncology.2007.10.010

[bib8] National Institute for Health and Clinical Excellence. Referral Guidelines for Suspected Cancer. Clinical Guideline 27. National Institute for Health and Clinical Excellence: London, 2005

[bib9] Neal DE, Donovan JL, Martin RM, Hamdy FC (2009) Screening for prostate cancer remains controversial. Lancet 374: 1482–1483 19664817 10.1016/S0140-6736(09)61085-0

[bib10] Neal RD, Allgar VL, Ali N, Leese B, Heywood P, Proctor G, Evans J (2007) Stage, survival and delays in lung, colorectal, prostate and ovarian cancer: comparison between diagnostic routes. Br J Gen Pract 57: 212–219 17359608 PMC2042569

[bib11] Pencavel TD, Strauss DC, Thomas GP, Thomas JM, Hayes AJ (2010) Does the two-week rule pathway improve the diagnosis of soft tissue sarcoma? A retrospective review of referral patterns and outcomes over five years in a regional sarcoma centre. Ann R Coll Surg Engl 92: 417–421 20487596 10.1308/003588410X12664192075972 PMC3180317

[bib12] Sandblom G, Varenhorst E, Rosell J, Löfman O, Carlsson P (2011) Randomised prostate cancer screening trial: 20 year follow-up. BMJ 342: d1539 21454449 10.1136/bmj.d1539 PMC3069219

[bib13] Schröder FH, Hugosson J, Roobol MJ, Tammela TL, Ciatto S, Nelen V, Kwiatkowski M, Lujan M, Lilja H, Zappa M, Denis LJ, Recker F, Berenguer A, Määttänen L, Bangma CH, Aus G, Villers A, Rebillard X, van der Kwast T, Blijenberg BG, Moss SM, de Koning HJ, Auvinen A (2009) Screening and prostate-cancer mortality in a randomized European study. N Engl J Med 360: 1320–1328 19297566 10.1056/NEJMoa0810084

